# HELLO: a protocol for a cluster randomized controlled trial to enhance interpersonal relationships and team cohesion among ICU healthcare professionals

**DOI:** 10.1186/s40635-024-00677-w

**Published:** 2024-10-07

**Authors:** Elie Azoulay, Nancy Kentish Barnes, Sheila Nainan Myatra, Maria-Cruz Martin Delgado, Yaseen Arabi, Carole Boulanger, Giovanni Mistraletti, Maria Theodorakopoulou, Vernon Van Heerden, José-Artur Paiva, Oktay Demirkýran, Gabriel Heras La Calle, Abdulrahman Al Fares, Gaston Burghi, Guy Francois, Anita Barth, Jan De Waele, Samir Jaber, Michael Darmon, Maurizio Cecconi

**Affiliations:** 1 Department of Intensive Care and Intensive Medicine, Paris-Cité University. Saint-Louis Hospital, APHP, Paris, France; 2 Department of Anaesthesiology, Critical Care and Pain, Tata Memorial Hospital, Homi Bhabha National Institute Mumbai, Mumbai, India; 3 Department Intensive Care Medicine Hospital 12 de Octubre. Research Institute “Hospital 12 de Octubre (imas12)”, Universidad Complutense de Madrid, Madrid, Spain; 4 Intensive Care Department, King Abdullah International Medical Research Center, King Abdulaziz Medical City, Ministry of National Guard Health - Affairs, and College of Medicine, King Saud Bin Abdulaziz University for Health Sciences, Riyadh, Saudi Arabia; 5 Royal Devon University NHS Foundation Trust, Barrack Road, Exeter, UK; 6 Dipartimento di Fisiopatologia medico-chirurgica e dei trapianti. A.S.S.T. Ovest Milanese, Università degli Studi di Milano, Ospedale Civile di Legnano, Legnano, MI Italy; 7 First Department of Critical Care and Pulmonary Diseases, Evangelismos General Hospital of Athens, National and Kapodistrian University of Athens, 10676 Athens, Greece; 8 Department of Anesthesiology, Critical Care and Pain Medicine, Faculty of Medicine, Hadassah Medical Center, Hebrew University of Jerusalem, Jerusalem, Israel; 9 Intensive Care Department, Centro Hospitalar Universitario S. Joao, Faculty of Medicine, University of Porto, Grupo Infecao e Sepsis, Porto, Portugal; 10 Department of Intensive Care, Cerrahpaşa Faculty of Medicine, Istanbul University-Cerrahpaşa, Istanbul, Turkey; 11 International Research Project for the Humanisation of Intensive Care Units, Proyecto HU-CI, Humanizing Healthcare Foundation. Intensive Care Unit, Hospital Universitario de Jaén, Jaén, Spain; 12 Department of Anesthesia, Critical Care Medicine, and Pain Medicine, Al-Amiri Hospital, Ministry of Health, Kuwait Extracorporeal Life Support Program, Al-Amiri Center for Respiratory and Cardiac Failure, Ministry of Health, Kuwait City, Kuwait; 13 Intensive Care Unit, Hospital Maciel, ASSE-Montevideo, Montevideo, Uruguay; 14 European Society of Intensive Care Medicine (ESICM), Brussels, Belgium; 15 Department of Intensive Care Medicine, Ghent University Hospital, Department of Internal Medicine and Pediatrics, Faculty of Medicine and Health Sciences, Ghent University, Ghent, Belgium; 16 Department of Anesthesia and Intensive Care unit, Regional University Hospital of Montpellier, St-Eloi Hospital, University of Montpellier, CEDEX 5, France; PhyMedExp, University of Montpellier, INSERM U1046, CNRS UMR, 9214 Montpellier, France; 17 Department of Biomedical Sciences, Humanitas University, Via Levi Montalcini, Pieve Emanuele, MI. 2IRCCS Humanitas Research Hospital, Via Manzoni 56, Rozzano, Milan, 20089 Italy; 18 Faculty of Health Sciences, Department of Nursing and Midwifery, University of Debrecen, Debrecen, Hungary

**Keywords:** Mental health, Nurses, Burnout, Psychology, Shortage

## Abstract

**Background:**

Mental health symptoms among healthcare professionals (HCP) in intensive care units (ICUs) are a significant concern affecting both HCP well-being and patient care outcomes. Cross-sectional studies among members of the European Society of Intensive Care Medicine (ESICM) report up to 50% burnout rates. Determinants of burnout include communication, team cohesion, psychological support, and well-being promotion. We designed the 'Hello Bundle' intervention to mitigate burnout among ICU-HCPs by fostering positive social interactions and a supportive work environment. This justification synthesizes evidence from social psychology, positive psychology, and healthcare communication research to support the intervention. The 'Hello Bundle' aims to enhance interpersonal relationships, improve team cohesion, and reduce burnout rates. The six components include: Hello campaign posters, email reminders, integrating greetings in morning huddles, hello jars, lead-by-example initiatives, and a daily updated hello board in each ICU. This protocol describes a cluster randomized controlled trial to evaluate the effectiveness of the intervention.

**Methods:**

This protocol describes a cluster randomized controlled trial (RCT) conducted among ESICM-affiliated ICUs, consisting of at least 73 clusters with in average of 50 respondents per cluster, totaling approximately 7300 participants. Intervention clusters will implement the 6-component Hello Bundle between October 14 and November 10, 2024, while control clusters will be wait-listed to receive the intervention in January 2025 after the RCT concludes. Clusters will be matched based on ICU size (fewer or more than 20 beds), region, and average 2023 mortality. The primary outcome is the proportion of HCPs with burnout between intervention and control clusters at the end of the intervention. Secondary outcomes include comparing the following between clusters: (1) number of HCPs with high emotional exhaustion; (2) number with high depersonalization; (3) number with loss of accomplishment; (4) perception of ethical climate (5) satisfaction at work (VAS); (6) professional conflicts; (7) intention to leave the ICU (VAS); (8) patient-centered care rating; (9) family-centered care rating. The last secondary outcome is the comparison of burnout rates before and after the intervention in the intervention cluster. Outcomes will be based on HCP reports collected within four weeks before and after the intervention.

**Discussion:**

This is the first large trial of healthcare communication, social, and positive psychology intervention among ICU-HCPs. It holds the potential to provide valuable insights into effective strategies for addressing burnout in ICU settings, ultimately benefiting both HCPs and patients.

*Trial registration*: This trial was registered on ClinicalTrials.Gov on June 18, 2024. Registration: NCT06453616.

**Supplementary Information:**

The online version contains supplementary material available at 10.1186/s40635-024-00677-w.

## Introduction

### Background and rationale

Burnout among healthcare professionals (HCPs), particularly prevalent in the high-stress environment of the intensive care unit (ICU), is characterized by emotional exhaustion, depersonalization, and a decreased sense of personal accomplishment [1–4]. This phenomenon not only profoundly impacts the well-being of ICU staff but has substantial consequences on the quality of care [5]. Burnout can lead to decreased job satisfaction, increased turnover rates, and difficulties in recruitment, exacerbating existing staff shortages in critical care settings [6]. The consequences of burnout extend beyond individual HCPs, affecting team dynamics, communication, and, ultimately, patient and family outcomes [7]. In the ICU, where quick decision-making and effective teamwork are paramount, burnout among staff members can compromise the quality of care delivered to critically ill patients [5]. Additionally, burnout may contribute to medical errors, decreased patient satisfaction, and heightened morbidity and mortality rates. Thus, addressing burnout in the ICU is essential not only for safeguarding the HCP’s well-being but also for ensuring optimal patient care and mitigating the challenges posed by staff shortages.

In the ICU, burnout among HCPs is often associated with various mental health symptoms such as anxiety, depression, moral distress, and post-traumatic stress disorder (PTSD) [8, 9]. Therefore, a comprehensive approach that encompasses prevention, recognition (Table 1), reversal, and the cultivation of resilience is required to address mental health symptoms in ICU-HCPs [10]. Prevention efforts aim to create a supportive work environment and promote individual well-being from the outset, thereby mitigating the risk of burnout [11–13]. Strategies may include fostering open communication, providing access to resources for stress management and coping skills, and promoting work-life balance through flexible scheduling and support programs. However, despite preventive measures, burnout may still occur. Therefore, it is crucial to recognize the signs and symptoms of burnout early on through regular screening and assessments [14]. By identifying individuals at risk, interventions can be implemented promptly to prevent further escalation. Moreover, once burnout has developed, efforts to reverse its effects are essential. This may involve targeted interventions such as counseling, coaching, and peer support, as well as organizational changes to address systemic contributors to burnout. Additionally, building resilience among healthcare professionals is paramount in combating burnout and promoting long-term well-being [15, 16]. Resilience training programs can equip individuals with the skills and strategies needed to cope with stress, bounce back from adversity, and thrive in demanding healthcare environments. By adopting a proactive approach encompassing prevention, recognition, reversal, and resilience-building, healthcare organizations can effectively address burnout and support the well-being of ICU staff, ultimately enhancing patient care outcomes and mitigating the impact of staff shortages.

**Table 1:** Common signs and symptoms associated with burnout among healthcare professionals

Signs of burnout	Description
Emotional exhaustion	Feeling emotionally drained, overwhelmed, and depleted of energy
Depersonalization	Developing negative, cynical attitudes and behaviors towards patients, colleagues, or work tasks
Reduced personal accomplishment	Experiencing a sense of ineffectiveness, low self-esteem, and diminished sense of achievement in one's work
Chronic fatigue	Persistent feelings of physical and mental fatigue, even after adequate rest
Increased irritability	Becoming easily frustrated, short-tempered, or impatient with colleagues, patients, or tasks
Difficulty concentrating	Struggling to focus, make decisions, or retain information, leading to decreased productivity and effectiveness
Withdrawal from work or social activities	Withdrawing from work-related tasks or social interactions, isolating oneself from colleagues and friends
Physical symptoms	Experiencing physical symptoms such as headaches, muscle tension, gastrointestinal issues, or changes in appetite or sleep patterns
Lack of motivation	Feeling apathetic, disengaged, and lacking enthusiasm or interest in work tasks or professional responsibilities
Increased Absenteeism	Taking more sick leave or absences from work than usual, often due to physical or mental health concerns related to burnout
Loss of empathy	Struggling to empathize with patients' or colleagues' experiences, emotions, or needs, leading to diminished quality of patient care

### ICU humanization and the aims of educational research

This project aims to encourage ICU personnel to adopt positive habits in their interactions with staff members. By disseminating the results in international scientific journals, we hope to enhance behavioral and communication skills among intensive care physicians and nurses, which are essential for the occupational well-being of healthcare workers.

The HELLO Trial seeks to drive cultural change in communication within ICU staff, creating a true partnership—a therapeutic alliance—to promote appropriate patient care, a positive work environment, and the psychological well-being of healthcare workers. As such, this protocol can be considered "educational research": it introduces best practices from a relational perspective among colleagues, while measuring their impact to gain scientific insights.

We believe that implementing the simple habits outlined in the HELLO Trial will help healthcare workers create a better ICU environment, potentially serving as a protective factor against burnout and helping staff members feel less isolated and stressed in their highly emotional daily work.

The new habits acquired during the HELLO Trial are intended to be sustained beyond the study's conclusion, maintaining the anticipated benefits. This study also provides intensivists the opportunity to create an international platform for idea exchange, with the potential to significantly improve the quality of planned interventions. If proven effective, this straightforward approach could serve as a model for ICUs worldwide.

### Design of the intervention

Few interventional studies have been conducted to prevent or mitigate burnout in HCPs. A systematic review and meta-analysis among physicians found that interventions were associated with only small reductions in burnout [17]. This study also suggested prioritizing organization-directed interventions that are delivered to experienced HCPs and in primary care. While mindfulness-based training was effective against burnout, it was ineffective in reducing anxiety or depression [18].

For this study, the dedicated steering committee from the European Society of Intensive Care Medicine (ESICM) aimed to design an intervention to reduce symptoms of burnout that would be feasible in ICUs globally and acceptable to ESICM members. Our steering committee considered four types of interventions: (1) organizational (staffing and workload management, work environment improvement through teamwork and communication, or training and development for resilience and professional growth); (2) individual (mindfulness and stress reduction through meditation and yoga, or health and wellness programs through physical fitness and healthy eating); (3) systemic (work-life balance policies, burnout awareness campaigns, mentorship programs, or peer support groups); and (4) technical (Electronic Health Records (EHR) optimization). As a teaching society aware of the devastating impact of burnout [19] and committed to prioritizing HCP well-being, maintaining a healthy workforce, and ensuring high-quality patient care [2], we selected an organizational intervention targeting the work environment (teamwork and communication). The "Hello Bundle" is a multi-faceted intervention designed to foster positive social interactions and a supportive work environment, through simple, cost-effective measures that are feasible in diverse global settings and structures.

### The "Hello Bundle": a 6-component intervention stemming from social psychology, positive psychology, and healthcare communication research

#### Theoretical framework

We aim to reshape HCP behaviors by reinforcing social norms and interactions within the ICU work environment. It is recognized that greetings and social niceties improve work atmosphere and climate, enhance emotional well-being and help build trust and cohesion among team members, which is essential for effective teamwork in high-stress environments like ICUs [20, 21]. The intervention also aims to influence how HCPs perceive their colleagues, give timely positive feedback, praise each other, and relate to each other. Additionally, we seek to change group dynamics in terms of conformity, leadership, and intergroup conflicts. The HELLO Bundle promotes positive emotions (happiness, joy, and contentment) and fosters individual strengths and virtues such as resilience, gratitude, and optimism. Positive emotions broaden individuals' thought-action repertoires, leading to greater resilience and reduced burnout [22]. We expect each component of the HELLO Bundle to enhance positive relationships, positively influence well-being and work satisfaction, and engage HCPs in activities that help achieve common professional goals [23]. Finally, effective communication is critical for successful teamwork in healthcare settings. Simple gestures like saying “Hello” can open channels for more meaningful communication, reducing misunderstandings and improving collaboration [24].

#### Intervention components and evidence

Hello campaign poster: will serve as visual cues (the posters), constant reminders of desired behaviors, reinforcing social norms and increasing the likelihood of engagement [25].

Email reminders: Twice weekly email reminders sent by the ESICM research staff act as behavioral prompts, encouraging consistent engagement with the intervention. This approach aligns with the principles of digital nudging [26].

Morning huddles: Incorporating greetings into morning huddles enhances team building and cohesion and sets a positive tone for the day. Morning huddles have been shown to improve communication and teamwork in healthcare settings [27].

Hello jar: The Hello Jar (Hello box) in which HCPs can leave messages for their colleagues provides a platform for positive reinforcement through recognition and appreciation, boosting morale and fostering a supportive work environment [28]. It can be seen as the Skinner box of modern times [29].

Lead by example: By using positive communication behavior, nursing and medical leaders can shape HCPs’ perceptions of the ICU and promote a stronger ICU climate. By greeting colleagues, they set a standard of model positive behavior for others to follow. Role modeling by leaders is crucial for shaping organizational culture [30].

Hello board: The Hello Board serves as an interactive tool for continuous engagement, promoting ongoing positive interactions and a sense of community among staff [11, 31].

#### Expected outcomes

Simple, low-cost interventions aimed at improving workplace interactions and social support can significantly reduce burnout among healthcare professionals [17], and improve job satisfaction by creating a positive work environment, leading to reduced turnover intentions [2, 32].

### Objectives of the HELLO trial

Our primary objective is to evaluate the impact of the HELLO bundle on the prevalence of burnout in ICU-HCPs. The primary outcome is the proportion of HCPs with post-intervention burnout between intervention and control clusters. One additional objective is the comparison of burnout rates before and after the intervention in the secondary outcomes include comparing the following between clusters: (1) number of HCPs with high emotional exhaustion; (2) number with high depersonalization; (3) number with loss of accomplishment; (4) perception of ethical climate (VAS); (5) satisfaction at work (VAS); (6) professional conflicts (VAS); (7) intention to leave the ICU (VAS); (8) patient-centered care rating (VAS); (9) family-centered care rating (VAS). intervention cluster.

The last secondary outcome is the comparison of burnout rates before and after the intervention in the intervention cluster. Outcomes will be based on HCP reports collected within four weeks before and after the intervention.

### Trial design

We designed a cluster RCT with a parallel design and 1:1 allocation ratio to evaluate the effectiveness of the HELLO bundle compared to a waitlist control group among at least 73 clusters of an average of 50 ICU-HCPs. Clusters will be matched based on ICU size (fewer or more than 20 beds) and country.

### Methods: participants, interventions, and outcomes

#### Study setting and eligibility criteria of the participating ICUs

An invitation to participate in the HELLO RCT, along with two reminders, was sent to all ESICM affiliates. Responses indicating interest in the trial were received from 679 individuals working in 679 centers. The respondents were asked to:

1. Identify a nurse–physician dyad who would act as local investigators for the trial.
2. Complete a form detailing the characteristics of their ICU.
3. Organize a unit-level meeting to provide information about the trial, including the type of intervention, the study timeline, and the nature of the data to be collected.
4. Attend one of three videoconferences (or watch the recording) that provided information on the study design, the HELLO bundle, the burnout instrument, and associated questions.
5. Obtain IRB approval and inform the local research department at each hospital.

**Fig. 1:**
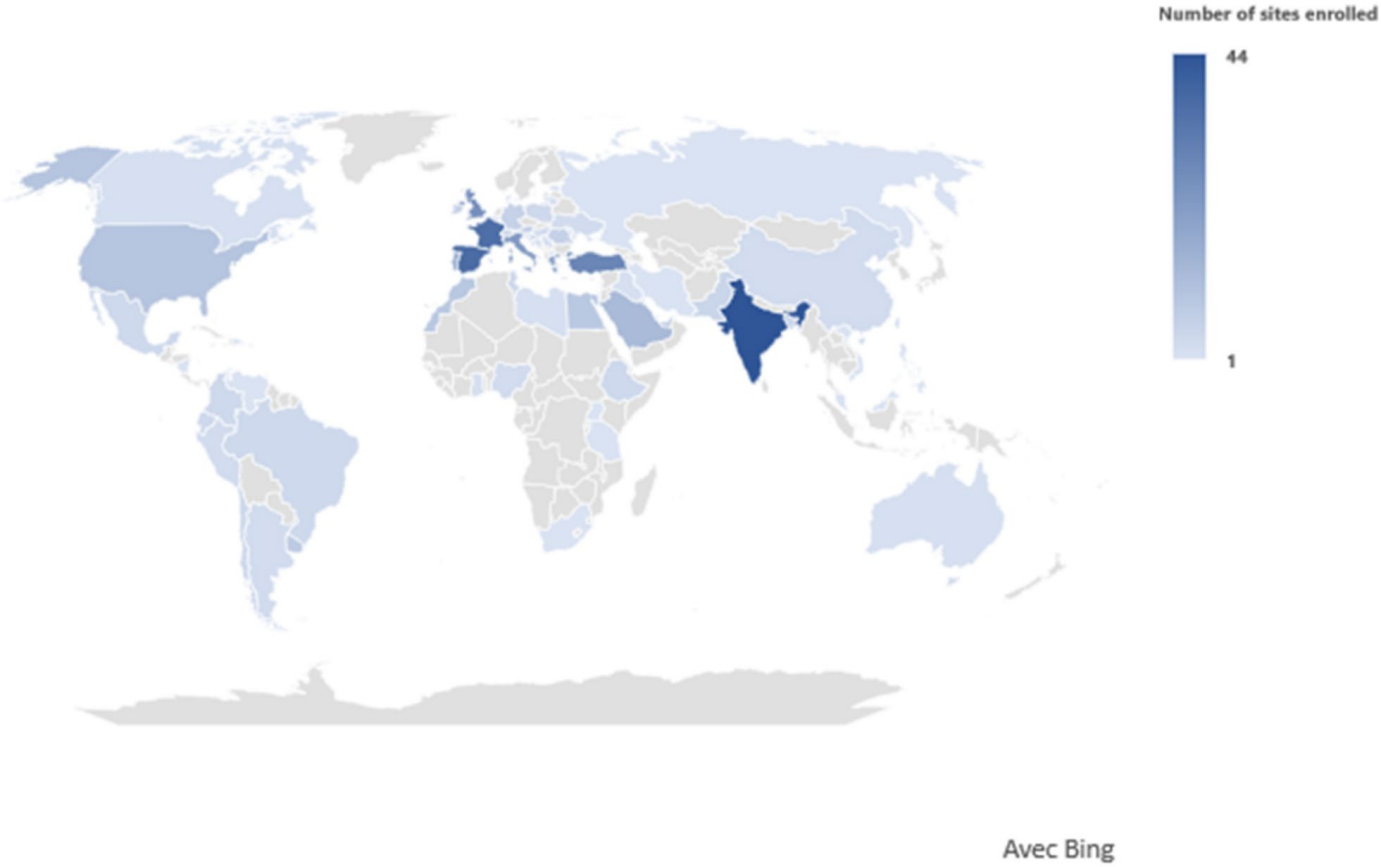
Map chart showing participating countries across geographical regions

**Fig. 2:**
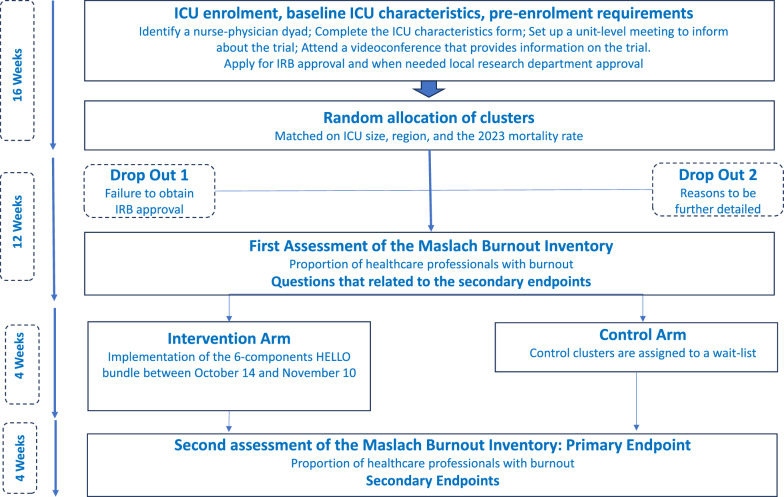
HELLO cluster randomized controlled trial flowchart procedures and timeline

Among the 679 centers that expressed interest in the trial, 434 completed the first four commitments and were subsequently randomized into either the intervention group (*n* = 217) or the control group (*n* = 217). Table 2 and Fig. 1 list the 434 ICUs that were randomized. As shown in Fig. 2, participation in the trial is contingent upon obtaining IRB approval, hence, we anticipated more centers than the 146 needed based on the sample calculation to avoid being underpowered.

**Table 2:** Participating countries

Region	Number of sites	Region	Number of sites
*Africa* • Egypt 9 • Ethiopia 4 • Ghana 2 • Libya 2 • Morocco 9 • Nigeria 3 • South Africa 1 • Tanzania 1 • Tunisia 4 • Uganda 1	33	*Southern Europe* • Bosnia and Herzegovina 2 • Croatia 7 • Greece 20 • Italy 23 • North Macedonia 1 • Portugal 17 • Serbia 2 • Slovenia 3 • Spain 37 • Turkey 29	141
*Asia* • Bangladesh 5 • China 3 • India 44 • Malaysia 1 • Pakistan 5 • Philippines 1 • Singapore 2 • Taiwan 3 • Vietnam 1	65	*Middle East* • Bahrain 1 • Iran 1 • Iraq 2 • Israel 4 • Kuwait 3 • Saudi Arabia 13 • United Arab Emirates 7	31
*Northern Europe* • Denmark 1 • Estonia 1 • Ireland 6 • Lithuania 2 • United Kingdom 26	36	*North America* • Canada 2 • Dominican Republic 1 • Mexico 4 • Nicaragua 1 • Puerto Rico 1 • United States 10	19
*Western Europe* • Austria 6 • Belgium 2 • France 36 • Germany 6 • Switzerland 6	56	*Oceania* • Australia 2	2
*Eastern Europe* • Hungary 3 • Moldova 1 • Poland 4 • Romania 6 • Russian Federation 1 • Ukraine 3	18	*South and Central America* • Argentina 3 • Brazil 4 • Chile 4 • Colombia 4 • Ecuador 4 • Peru 3 • Uruguay 8 • Venezuela 1	31

#### Modalities of informed consent

Healthcare professionals (HCPs) participating in this trial must agree to be part of the study and consent to having their data published once they log into the REDCap file, which will collect the Maslach Burnout Inventory (MBI), secondary outcomes, and demographic data. Additionally, some countries may require individual informed consent to be signed by each HCP prior to trial participation.

### Intervention

#### Explanation for the choice of comparators

This study compares intervention clusters to waitlist control clusters. We relied on a central randomization to select which clusters receive the intervention now and which are assigned to a waitlist to receive the intervention after the RCT concludes, in January 2025. Through utilizing a waitlist design, we expect that placebo effects will be effectively balanced between groups, as the control group’s knowledge of soon receiving a supportive intervention may yield improvements in their outcomes [33].

#### Intervention description

Intervention clusters will implement the HELLO bundle for four weeks, starting Monday, October 14, 2024. Twenty posters will be displayed in various locations within the ICU, including workstations, dressing rooms, and toilets. These posters, designed by the ESICM steering committee, will be shipped to all participating centers in either August (for the intervention group) or January (for the control, waitlist centers). The blue posters feature the term "HELLO" in different languages, the HELLO logo with a smiley face for the letter "O", and the ESICM logo.

Every Monday and Friday during the four-week intervention, the study's ESICM coordinators will send emails to the HELLO dyad at each participating center, highlighting the impact of positive psychology, team cohesion, and communication on healthcare professionals' (HCPs) well-being. These emails will then be forwarded to all HCPs via email, WhatsApp, or any other channel, and may also be displayed in the ICU by the local dyad. Saying "HELLO" before any huddles, handovers, or staff meetings will be encouraged.

Two HELLO jars/boxes will be placed in the ICU, designed by ESICM and sent to the centers. HCPs will be invited to write and insert positive messages about their colleagues and their work experience in the ICU into these jars/boxes. These messages will be accessible to anyone in the ICU who wishes to read them. At least twice a week, the HELLO dyad (or the head nurse and the department chief) will visit the ICU to greet everyone, set an example, explain the study, and share their reasons for participating in the trial.

Lastly, two paperboards will be displayed and updated as needed. These boards can include direct messages, stickers, drawings, photos, or any positive notes directed to other HCPs or the entire ICU.

In the ICUs from the intervention cluster, ESICM officers will make contact once a week to assess a rough estimate of the intervention's dosage. Each of the six components will be scored from 0 to 4, as illustrated in Table 3.

**Table 3:** Adhesion score to measure the rate of implementation of the HELLO intervention

Adhesion score	0	1	2	3	4	+ 1
Number of Hello posters displayed (over 4 weeks)	None	< 5	5–10	10–15	> 15	More poster printed and displayed
Email reminders	Never forwarded	Forwarded only one week	Forwarded only two weeks	Forwarded only three weeks	Forwarded all the four weeks	Emails printed and displayed in the ICU
Hello during the Morning Huddles	Never	Rarely	Sometimes	Almost all the time	Always	Hello also introduced in other ICU meetings
Number of messages in the two Hello Jars	< 5	5–10	10–20	20–30	30–50	> 50
Lead by Example	Nurse and/or Physician never appeared in the ICU to say hello and promote the study	Nurse and/or Physician of the dyad came the first week to say hello and promote the study	Nurse and/or Physician of the dyad came the first two weeks to say hello and promote the study	Nurse and/or Physician of the dyad came the first three weeks to say hello and promote the study	Nurse and/or Physician of the dyad came all the four weeks to say hello and promote the study	Nurse and physician of the dyad set up specific meetings to explain and promote the study and took advantage of the study to promote well-being, team cohesion and communication
Number of messages in the two Hello Boards	< 5	5–10	10–20	20–30	30–50	> 50

#### Criteria for discontinuing or modifying allocated interventions {11b}

All participants will be informed that they may stop participating in the HELLO trial at any time and stop completing the questionnaires. However, as the study is fully anonymous we will not be able to remove their data from the global database.

#### Strategies to improve adherence to interventions and data collection

Five elements have been proposed to facilitate adherence to the interventions: (1) detailed communications: Emails and slides have been meticulously prepared to inform the investigators and enable them to inform their teams effectively; (2) centralized materials: all materials are centrally prepared and shipped to the participating ICUs; (3) instructional video: a 3-min video has been created to detail the implementation of the HELLO bundle. Although the video is in English, it uses non-verbal communication to illustrate the different components of the intervention, making it accessible to a wider audience; (4) Maslach Burnout Inventory (MBI): the MBI-HSS questionnaires for healthcare professionals are available in 18 different languages using validated versions provided by Mind Garden (https://www.mindgarden.com/117-maslach-burnout-inventory-mbi); (5) data collection: this study does not include any case report forms (CRFs). No data on patients or family members will be collected. Only the primary and secondary outcomes will be recorded in RedCap.

#### Relevant concomitant care permitted or prohibited during the trial

We have no restrictions on participants receiving other communication, mental health, psychosocial, and any support during our study.

### Outcomes

#### Primary endpoint

The proportion of HCPs with symptoms of burnout will be compared between the interventional and the control cluster. Symptoms of burnout will be measured using the validated version of the 22-item Maslach Burnout Inventory (MBI, Human Services version) [6, 7], which includes three subscales: emotional exhaustion (9 items), depersonalization (5 items), and personal accomplishment (8 items). Each item is scored from 0 (never) to 6 (every day). Respondents with high emotional exhaustion (≥ 27) and/or high depersonalization (≥ 10) scores will be considered to have symptoms of burnout [2].

#### Secondary endpoints

Secondary endpoints include comparing the following between clusters:

1. Emotional exhaustion subscale and the number of HCPs with high emotional exhaustion (score ≥ 27).
2. Depersonalization subscale and the number of HCPs with high depersonalization (score ≥ 10).
3. Loss of accomplishment subscale.
4. Perception of ethical climate (VAS).
5. Satisfaction at work (VAS).
6. Professional conflicts (VAS).
7. Intention to leave the ICU (VAS).
8. Patient-centered care rating (VAS).
9. Family-centered care rating (VAS).
10. Comparison of the proportion of HCPs with symptoms of burnout before and after the intervention in the intervention cluster.

Visual analogue scales (VAS) will be used to assess the intensity of unidimensional measures. Two anchors will be provided: for 0 (no symptom/lowest rating) and 10 (the most intense symptom/highest rating). VASs are convenient, easy, and rapid to administer and have been proven reliable for measuring a characteristic, subjective phenomenon, or attitude that is believed to range across a continuum of values and cannot easily be directly measured.

#### Participant timeline

As shown in Table 4 and Fig. 2, HCPs from both clusters will complete the MBI in September and in November 2024, while the HELLO bundle will be implemented in the interventional clusters between October 14 and November 10. No follow up will be set up for the participating HCPs.

**Table 4:** HELLO timeline figure

Timepoint	Study period			
	Enrollment	Pre-intervention	Intervention	Post-intervention
Completion of the ICU characteristics form	X			
Identification of the nurse–physician dyad	X			
Information of the ICU team	X			
Randomization of the ICUs	X			
IRB approval	X			
Approval by the hospital research department	X			
Intervention HELLO bundle (intervention arm) Wait list (control arm)			X X	
Informed consent		X		X
Demographic information		X		X
Assessments Maslach Burnout Inventory (primary endpoint) ¥ 12 additional questions (secondary endpoints) ¥		X X		X X

#### Sample size

**Fig. 3:**
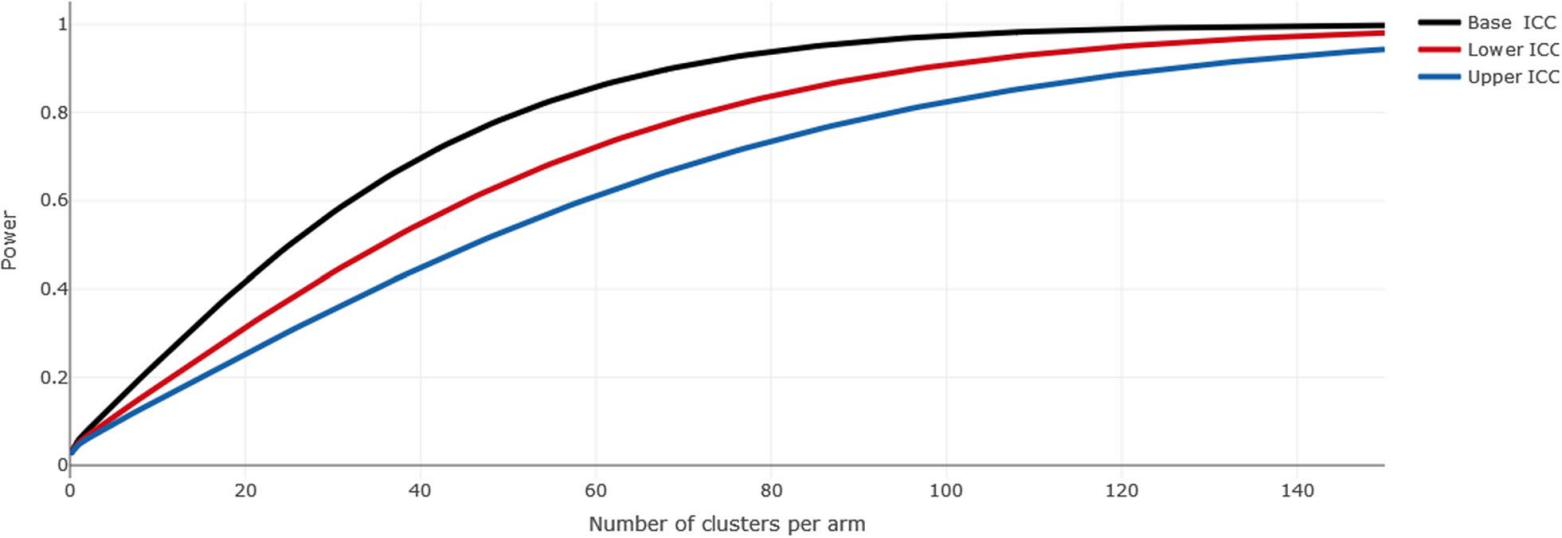
Sample size estimation assuming 50 respondents per cluster, a reduction in the prevalence of burnout from 39 to 30%, assuming an intra-class coefficient of 0.15 (base intra-class coefficient, range 0.1–0.2)

Statistical analysis will compare outcomes between intervention and control groups, aiming to demonstrate a reduction in the prevalence of burnout from 39 to 30%, assuming an intra-class coefficient of 0.15, and aiming for a statistical power of 80% (Fig. 3). Intra-class coefficient for the outcome variable was approximated and liberal when compared to precedent cluster trials performed on burnout performed in other context [34].

Primary and secondary analyses will be performed at an individual level adjusting for clustering. No imputation will be performed for missing variables.

Subgroup analyses will be conducted to explore potential moderators of intervention effectiveness.

Assuming an intra-class correlation of 0.15, 146 centers and 50 respondents per center, the study would allow to demonstrate a reduction in burnout prevalence from 39 to 30% with a statistical power of 80%. This study will enroll at least *k* = 73 clusters of 50 participants in average, totaling approximately *n* = 7300 participants (assuming an average cluster size of *m* = 50). The sample size was determined by power calculations from previous cross sectional studies [3, 7, 35, 36]. Based on estimated effect sizes, ICCs, and SDs of the outcome in the population from previous data, and accounting for attrition related to failure to obtain IRB or local research committees approval, we estimated that at least *k* = 73 clusters and *n* = 7300 participants would be sufficient to detect significant effects with 80% power in the primary outcome. However, because there was an uncertainty about the proportion of IRB and local research committees’ failure, as well as the number of ICUs finally not willing to participate to the trial, we included all the 434 ICUs (217 per group) who applied to the IRB before July 1st, 2024.

#### Assignment of interventions: allocation

Sequence generation: Cluster randomization matched on the ICU size and country has been performed using cvrand package in R.

Concealment mechanism: Randomization did not occur before IRB could be obtained in every center. However, as we randomized 217 clusters for a target of “at least” 73 clusters, all matched clusters have been randomized together—which ensures allocation concealment is maintained, limiting selection bias.

Implementation: Cluster were randomized to treatment or control within each strata with a 1:1 allocation ratio.

Assignment of interventions: blinding: Blinding for the HELLO trial is not possible.

### Statistical methods

#### Primary and secondary outcomes

Data will be described as median and interquartile range (IQR) or number and percentage. Categorical variables will be compared using Fisher's exact test and continuous variables using the nonparametric Wilcoxon test, Mann–Whitney test, or Kruskal–Wallis test.

The primary method of analysis will be a generalized linear mixed-effects model, which will be adjusted for cluster. We will use an intention-to-treat approach with a binary exposure variable indicating if the participant was randomized to the intervention or control group.

Predefined subgroup analyses will include:

1. Center and country effect as assessed using mixed effect model. Briefly, we will assessed effect of the intervention while accounting for center and country effect. Center and country will be examined in dedicated mixed effect model and will be added as random effect on the intercept. Impact of the intervention will be reported when adjusted for these confounders. Center and country effect will be reported [37]. To test for the significance of center and country effects on outcome, we will use permutation tests [38]. Difference in rate of burnout will across centers and countries will be reported as median odds-ratio with their confidence interval. Influence of centers and country will be plotted.
2. In addition, influence of adherence to protocol will be assessed. Impact of adherence on outcome will be reported by quartile of adherence.
3. Last, influence of the job of respondents will be assessed.

If any additional exploratory or sensitivity analyses should be performed, they will be reported in the manuscript as post hoc analyses and interpreted as exploratory hypothesis generating analyses.

We will use an appropriate bootstrapping method with an identity link. We will use two-tailed tests for all models with statistical significance thresholds of 0.05. Results will be reported as mean difference, incidence rate ratio or odds ratios as appropriate.

Statistical analyses will be performed with R statistical software, version 3.4.3 (available online at http://www.r-project.org/) and packages ‘lme4’,and’lmerTest’. A *p* value < 0.05 will be considered significant.

Results will be reported in adherence with standards for reporting implementation studies of complex interventions guidelines [39].

### Ethics

This study will not collect any data about patients or family members.

The study protocol will be reviewed and approved by the Institutional Review Board (IRB) or Ethics Committee of each participating institution according to country rules, ensuring compliance with ethical principles and guidelines for research involving human subjects. Informed consent will be obtained from all healthcare professionals (HCP) participating in the study as the first page of the survey requires that the respondent confirms her/his willingness to be part of the trial, emphasizing voluntary participation, and confidentiality.

In detail, every healthcare provider clicking on the link to complete burnout assessment and associated questions will have to tick two boxes: 1/ that she/he agrees to participate to this fully anonymous study and 2/ that she/he agrees that the provided information can be used for analyses and publication. The accompanying text will ensure healthcare professionals about the confidentiality of data. No data will be made available to centers.

Sensitive data will not be collected (ethnicity, religion, religiosity, etc.)

The database will be declared to the CNIL in France (the methodologist is Prof Darmon in France).

Measures will be taken to protect the privacy and confidentiality of participant data, including the use of secure electronic data storage and encryption methods where necessary. The study will be conducted in accordance with the principles outlined in the Declaration of Helsinki and other relevant ethical standards. Additionally, efforts will be made to disseminate the study findings in a transparent and timely manner, contributing to the body of knowledge on burnout prevention and intervention strategies in intensive care unit (ICU) settings.

### Confidentiality

Participants will not provide any identifying information (such as full names). Therefore, we will not collect any identifying information beyond what is sufficient for a unique ID (in addition to age and gender of the respondent).

### Plans for collection, laboratory evaluation, and storage of biological specimens for genetic or molecular analysis in this trial/future use

Not applicable. This study will not collect, evaluate, or store any biological specimens.

## Supplementary Information

Supplementary Material 1.

## Data Availability

All de-identified data and study materials will be made freely available after the study. However, regarding the trial data, no individualized data will be shared and only aggregated data will be available for sharing.
