# Correction: HELLO: a protocol for a cluster randomized controlled trial to enhance interpersonal relationships and team cohesion among ICU healthcare professionals

**DOI:** 10.1186/s40635-024-00702-y

**Published:** 2025-01-07

**Authors:** Elie Azoulay, Nancy Kentish Barnes, Sheila Nainan Myatra, Maria-Cruz Martin Delgado, Yaseen Arabi, Carole Boulanger, Giovanni Mistraletti, Maria Theodorakopoulou, Vernon Van Heerden, José-Artur Paiva, Oktay Demirkýran, Gabriel Heras La Calle, Abdulrahman Al Fares, Gaston Burghi, Guy Francois, Anita Barth, Jan De Waele, Samir Jaber, Michael Darmon, Maurizio Cecconi

**Affiliations:** 1 Department of Intensive Care and Intensive Medicine, Paris-Cité University. Saint-Louis Hospital, APHP, Paris, France; 2 Department of Anaesthesiology, Critical Care and Pain, Tata Memorial Hospital, Homi Bhabha National Institute Mumbai, Mumbai, India; 3 Department Intensive Care Medicine Hospital 12 de Octubre;. Research Institute “Hospital 12 de Octubre (imas12)”, Universidad Complutense de Madrid, Madrid, Spain; 4 Intensive Care Department, King Abdullah International Medical Research Center, King Abdulaziz Medical City, Ministry of National Guard Health, Affairs, and College of Medicine, King Saud Bin Abdulaziz University for Health Sciences, Riyadh, Saudi Arabia; 5 Royal Devon University NHS Foundation Trust, Barrack Road, Exeter, UK; 6 Dipartimento di Fisiopatologia, Medico-Chirurgica e dei Trapianti, A.S.S.T. Ovest Milanese, Università Degli Studi di Milano, Ospedale Civile di Legnano, Legnano, MI Italy; 7 First Department of Critical Care and Pulmonary Diseases, Evangelismos General Hospital of Athens, National and Kapodistrian University of Athens, 10676 Athens, Greece; 8 Department of Anesthesiology, Critical Care and Pain Medicine, Faculty of Medicine, Hadassah Medical Center, Hebrew University of Jerusalem, Jerusalem, Israel; 9 Intensive Care Department, Centro Hospitalar Universitario S. Joao, Faculty of Medicine, University of Porto; Grupo Infecao e Sepsis, Porto, Portugal; 10 Department of Intensive Care, Cerrahpaşa Faculty of Medicine, Istanbul University-Cerrahpaşa, Istanbul, Turkey; 11 International Research Project for the Humanisation of Intensive Care Units, Proyecto HU-CI; Humanizing Healthcare Foundation. Intensive Care Unit, Hospital Universitario de Jaén, Jaén, Spain; 12 Department of Anesthesia, Critical Care Medicine, and Pain Medicine, Al-Amiri Hospital; Kuwait Extracorporeal Life Support Program, Al-Amiri Center for Respiratory and Cardiac Failure, Ministry of Health, Kuwait City, Kuwait; 13 Intensive Care Unit, Hospital Maciel, ASSE, Montevideo, Uruguay; 14 European Society of Intensive Care Medicine (ESICM), Brussels, Belgium; 15 Department of Intensive Care Medicine, Ghent University Hospital. Department of Internal Medicine and Pediatrics, Faculty of Medicine and Health Sciences, Ghent University, Ghent, Belgium; 16 Department of Anesthesia and Intensive Care Unit, Regional University Hospital of Montpellier, St-Eloi Hospital, University of Montpellier, CEDEX 5, France; PhyMedExp, University of Montpellier, INSERM U1046, CNRS UMR, 9214 Montpellier, France; 17 Department of Biomedical Sciences, Humanitas University, Via Levi Montalcini, Pieve Emanuele, MI; 2IRCCS Humanitas Research Hospital, Via Manzoni 56, 20089 Rozzano, Milan Italy; 18 Faculty of Health Sciences, Department of Nursing and Midwifery, University of Debrecen, Debrecen, Hungary

**Correction: Intensive Care Medicine Experimental (2024) 12:90** 10.1186/s40635-024-00677-w

Following publication of the original article [1], it came to the journal's attention that because of a technical error during production of the article, the following affiliations had each been divided into two separate (incorrect) affiliations: affiliations 3, 4, 9, 11, 12, 15, 16, 17. As a result of this duplication, the affiliations information of the article was partly incorrect. The affiliations have since been corrected in the original article and the corrected affiliations information can be seen in this erratum; please refer to this corrected information. The publisher thanks you for reading this erratum and apologizes for any inconvenience caused.
