# Supplementary material for: HELLO: a protocol for a cluster randomized controlled trial to enhance interpersonal relationships and team cohesion among ICU healthcare professionals
Source: Intensive Care Med Exp. 2024 Oct 7;12:90. doi: 10.1186/s40635-024-00677-w (PMC11459960; doi:10.1186/s40635-024-00677-w)
Supplement: Supplementary file 1 — Supplementary Material 1. [file 40635_2024_677_MOESM1_ESM.docx]

**Collaborators**

| **Investigator 1** | | **Investigator 2** | | **Center** | **Localisation** | |
| --- | --- | --- | --- | --- | --- | --- |
| **First name 1** | **Last name 1** | **First name 2** | **Last name 2** | **Hospital** | **City** | **Country** |
| Carolina | Bastos | Carolina | Bastos | Hospital perrando | Resistencia | Argentina |
| Agustin | Manchado Bruno | Daniela | López | Instituto de Trasplante y Alta Complejidad (ITAC) | Buenos Aires | Argentina |
| Agustin | Manchado Bruno | Lucas | Romano | Sanatorio Mater Dei | Buenos Aires | Argentina |
| Gordon | Flynn | Rachel | Longhurst | North Canberra Hospital ICU | Canberra | Australia |
| Joannidis | Michael | Adrigan | Erwin | University Hospital | Innsbruck | Austria |
| hossam | osman | hossam | osman | salmanyia medical complex | Manama | Bahrain |
| Lutful | Aziz | julia | parvin | Evercare Hospital Dhaka | Dhaka | Bangladesh |
| Md Tariqul | Islam | Md Tariqul | Islam | Dhaka Medical College Hospital | Dhaka | Bangladesh |
| Mohammad MUFIZUL Islam | Polash | Mohammad Mufizul Islam | Polash | Square Hospital | Dhaka | Bangladesh |
| Rajib | Hasan | Khayrul | Basher | Asgar Ali Medical College and & Hospital | Dhaka | Bangladeshi |
| Zoé | Pletschette |  |  | Hôpital Erasme | Brussels | Belgique |
| Gilberto | Friedman | Karina | Azzolin | Hospital de Clínicas de Porto Alegre | Porto Alegre | Brazil |
| Hugo | Paiva | Anna Grazielle | Gadelha | Hospital Promater | Natal | Brazil |
| Sandra | Dial | Noel | Dowell | McGill University Health Centre | Montreal | Canada |
| Magdalena | Vera | Gustavo | Zambrano Fuentes | Hospital Clinico de la Red de Salud UC-CHRISTUS | Santiago | Chile |
| Pui Ning Pauline | Yeung | Peter Chi Keung | Lai | Queen Mary Hospital | Hong Kong | China |
| Jorge Iván | Alvarado Sánchez | Jhon Alvaro | Niño Aponte | Fundación Santa Fe de Bogotá | Bogotá | Colombia |
| Dormar David | Barrios Martínez | Maria Isabel | Sierra Henao | Hospital Universitario San Vicente Fundación | Medellin | Colombia |
| SRĐAN | VRANKOVIĆ | NIKOL | ABDURAHMANOVIĆ | OB šibensko-kninske županije | Šibenik | Croatia |
| Sanda | Stojanovic Stipic | Tina | Kuseta | University Hospital of Split | Split | Croatia |
| Maja | Karaman Ilic | Monika | Horvat | Radiochirurgia | Zagreb | Croatia |
| Stella | Davila Šarić | Nataša | Goričan | University Hospital Centre Zagreb | Zagreb | Croatia |
| Morten | Bestle | Sanne thers | Lauritzen | Nordsjaellands Hospital | Hillroed | Denmark |
| Rommell | Morel | Zurinlleli | Espinal | Hospital Metropolitano de Santiago | Santiago de los Caballeros | Dominican Republic |
| Francisco Javier | Cordova Loor | Alisson Dayhana | Cadena Velarde | Hospital General IESS Babahoyo | Babahoyo | Ecuador |
| Alexandra | Saraguro | Paulina | Gutierrez | Hospital General Latacunga | Latacunga | Ecuador |
| DIEGO | MOROCHO TUTILLO | ANDREA | PEÑA | HOSPITAL DE ESPECIALIDADES "EUGENIO ESPEJO" | Quito | Ecuador |
| Tarek | Hemaida | Yousef | Abdulghany | Aswan University Hospital | aswan | Egypt |
| Eman Ibrahim El-Desoki | Mahmoud | Khadega | Mahmoud Hussien | National Hepatology and Tropical Medicine Reseaech institute | Cairo | Egypt |
| Eleia | Mosaad | Hadeer | Khedr | Aswan Heart Centre - Magdi Yacoub Foundation | Aswan | Egypt |
| Eleia | Mosaad | Hadeer | Khedr | Aswan Heart Centre - Magdi Yacoub Foundation | Aswan | Egypt |
| Saad | Moharam | Abdurrahman | Shuaib | Tanta university hospital | Tanta | Egypt |
| Kyrillos | Nassim | Yasmeen | Ahmed | Nasser Institute Hospital for Research and Treatment | Cairo | Egypt |
| Wael | Kassem | Kamilia | Samy | Sharq Elmadina Hospital | Alexandria | Egypt |
| Shewit | Weldegergs | Mulugeta | Belay | Ekakotebe General Hospital | Addis Ababa | Ethiopia |
| morel | jerome | cuerq | marion | CHU St etienne | Saint-Etienne | France |
| belaid | BOUHEMAD | agnes | Geantot | CHU Dijon | Dijon | France |
| Alexandre | Demoule | Auxane | Paradot | La Pitié-Salpêtrière | Paris | France |
| Anne | SAGNIER | Fabienne | HELOISE | Centre Hospitalier Simone Veil | BEAUVAIS | France |
| Albert | CAO | Laura | Miranda | Centre hospitalier intercommunal Meulan Les Mureaux | Meulan en Yvelines | France |
| Élie | Azoulay | Élie | Azoulay | Hôpital Saint-Louis | Paris | France |
| KAWTAR | RGHIOUI | mael | philippe | HOPITAL SIMONE VEIL | EAUBONNE | France |
| Juliette | Audibert | Gaëtan | Badre | CH Louis Pasteur | Le Coudray | France |
| OLIVIER | GUISSET | vaNESSA | alves | Hôpital Saint André- CHU Bordeaux | Bordeaux | France |
| LAURENT | LAINE | LAURENCE | ROBIN | CENTRE HOSPITALIER D'ANGOULEME | Angouleme | France |
| SAMIR | JABER | Jeanne | BOYER | Hôpital Saint Eloi | Montpellier | France |
| Laurent | ARGAUD | Marion | PROVENT | HOSPICES CIVILS DE LYON - HOPITAL EDOUARD HERRIOT | Lyon | France |
| CYRIL | GOULENOK | NAJIB | JABER | hopital Privé Jacques Cartier | MASSY | France |
| Olivier | Duranteau | Caroline | Touzet | HNIA Percy | Clamart | France |
| CHARLOTTE | QUENTIN | christine | Poulain | Centre hospitalier de Saint Malo | Saint malo | France |
| ERIC | DELPIERRE | DELPHINE | LANDRY | GHEF - GRAND HOPITAL DE L'EST FRANCILIEN | JOSSIGNY | France |
| Djillali | Annane | Ségolène | Jourdier | Raymond Poincaré | Garches | France |
| Anne Astrid | Bourion | Muriel | Tanguy | Hopital de cholet | Cholet | France |
| Yassir | AARAB | Solene | ARBET | Clinique Saint Jean Sud de France | Saint Jean de Vedas | France |
| Marion | Monnin | Emmanuelle | Dos Santos | centre hospitalier de beziers | beziers | France |
| Pierre | COUHAULT | Angelina | Barrage | Gabriel Montpied Hospital | Clermont-Ferrand | France |
| Jörg | Reutershan | Verena | Krumsdorf | Klinikum Bayreuth | Bayreuth | Germany |
| Alexander | Dejaco | Silke | Auer | University Hospital Regensburg | Regensburg | Germany |
| Christian | Siebers | Kathrin | Schüle | Klinikum GAP | Garmisch-Partenkirchen | Germany |
| Oluwayemisi Esther | Ekor | Sarah | Amoo | Cape Coast Teaching Hospital | Cape Coast | Ghana |
| PANAGIOTA | STAMOU | MARIA | VACHLA | General Hospital of Halkida | Halkida | Greece |
| SOPHIA ANASTASIA | MOURATOGLOU | EFI | STRATI | IPPOKRATEIO GENERAL HOSPITAL | THESSALONIKI | Greece |
| Marina | Oikonomou | CHRYSANTHI | PERROU | Kateríni General Hospital | Kateríni | Greece |
| Stavros | Mantzoukis | Asterios | Theodosiou | General Hospital of Ioannina, Hatzikosta | Ioannina | Greece |
| Ioannis | Andrianopoulos | Maria | Markou | University Hospital of Ioannina | Ioannina | Greece |
| ELENI | PALLI | THEODORA | ZADE | University Hospital of Larissa | Larissa | Greece |
| Eleni | Volakli | Alexia | Tamouridou | University Hospital of Larisa | Larissa | Greece |
| ZAFEIRIA | MASTORA | HELEN | TOMPROU | Thoracic Diseases General Hospital "Sotiria" | athens | Greece |
| Anastasios | Sakkalis | Antonia | Bakali | Konstantopouleio | Athens | Greece |
| Schnur | Janos | Siko-Laky | Ivett | Heim Pal National Pediatric Institute | Budapest | Hungary |
| Ashish | Bindra | Charulata | Vinod Kumar | Jai Prakash Narain Apex Trauma Center, All India Institute of Medical Sciences | New Delhi | India |
| Sukhyanti | Kerai | Anita | Toppo | Maulana Azad Medical College and associated Lok Nayak hospital | New Delhi | India |
| Kishore | Mangal | Kamal | Prajapati | Fortis hospital | Jaipur | India |
| Raman | muraleedharan | Elizabeth | Varkey | Baby memorial hospital | Trivandrum | India |
| Amith | Narayan | Bindhu | R | Azeezia institute of medical sciences and research institute | Kollam | India |
| Ravi | Sreekumaran Nair | Shiva | Vasanthakumari Nagappan | SK Hospital | Thiruvananthapuram | India |
| Amit Kumar | Dey | Amit Kumar | Dey | IMS and SUM Hospital Campus 2 | Bhubaneswar | India |
| GAURAV | ACHARYA | JIJO | VARUGHESE | BHOPAL MEMORIAL HOSPITAL AND RESEARCH CENTRE | Bhopal | India |
| SUBHAL | DIXIT | SWATI | KANADE | SANJEEVAN HOSPITAL | PUNE | India |
| Dr ziyokov | Joshi | Dr ziyokov | Joshi | Tagore hospital and heart care | Jalandhar | India |
| Santosh | Singh | Ch Vamshi | Krishna | Care hospitals | Hyderabad | India |
| Bhavna | Gupta | Vishnu | Y | All India Institute of Medical Sciences | Uttarakhand | India |
| Lakshmikanthcharan | Saravanabavan | Muneshwari | Govindharaj | Royalcare superspeciality hospital | Coimbatore | India |
| Sheila | Myatra | Gorade | Manoj | Tata Memorial Hospital | Mumbai | India |
| Rajesh | Pande | Rajesh | Pande | BLK MAX Superspeciality Hospital | New Delhi | India |
| LALIT | GUPTA | Hanuman | Barodiya | Lok Nayak Hospital | Delhi | India |
| Hakim irfan | Showkat | DAR | SUHAIL | SRINAGAR MEDCITY HEART INSTITUTE | SRINAGAR | India |
| Alisha | Chaudhury | Suchismita | Behera | SUM ULTIMATE MEDICARE | Bhubaneswar | India |
| RISHI | Katiyar | Robin | Mahor | Apollo Sage hospital | Bhopal | India |
| Shilpushp | Bhosale | Gorade | Manoj | ACTREC | Navi Mumbai | india |
| Junaid | Hashmi | Adeline | Milne | Our Lady of Lourdes Hospital | Drogheda | Ireland |
| Michelle | Duggan | Fiona | O'Shea | Mayo University Hospital | Castlebar | Ireland |
| Paolo | Formenti | Giovanni | Brenna | ASST NORD MILANO, OSPEDALE BASSINI | CINISELLO BALSAMO | Italy |
| Rosanna | Vaschetto | Natalia | Gentile | Maggiore della carita’ | Novara | Italy |
| Annalisa | Piccolo | Marzia | Martino | Grande Ospedale Metropolitano "Bianchi Melacrino Morelli" | Reggio di Calabria | Italy |
| FRANCESCO | DE LAZZARO | SOFIA | TABORRI | POLICLINICO UMBERTO 1 | Roma | Italy |
| Antonella | Cotoia | Grazia | Lombardi | Azienda Universitario Ospedaliero di Foggia | Foggia | Italy |
| Annachiara | Marra | Giovanni | De Martino | Azienda Ospedaliera Universitaria 'Federico II' | Naples | Italy |
| Maria Grazia | Bocci | Elena | Mattiucci | Istituto Nazionale per le Malattie Infettive L. Spallanzani IRCCS Roma | Rome | Italy |
| Simone | Piva | Lorenzo | Pilati | ASST Spedali Civili di Brescia | Brescia | Italy |
| Stefano | Romagnoli | Carolina | Forcinti | Azienda Ospedaliero-Universitaria Careggi | Florence | Italy |
| Luca | Montini | Serena | Lochi | Fondazione Policlinico Universitario Agostino Gemelli, IRCCS | Roma | Italy |
| Elisabetta | Cerutti | Nadia | Moroni | Azienda Ospedaliero Universitaria delle Marche | Ancona | Italy |
| Anup | Varghese | Ghasoom | Ghasoom | Chest diseases hospital | Kuwait City | Kuwait |
| Hana | Yahya | Mohammed | Eltoncy | Zliten medical centre | Zliten | Libya |
| Neringa | Balciuniene | Ingrida | Urboniene | The Hospital of Lithuanian University of Health Sciences Kauno klinikos | Kaunas | Lithuania |
| Amelia | Mohamed Ain | Li Li | Kok @ Khor | Hospital Sultan Abdul Halim | Sungai Petani, Kedah | Malaysia |
| Pavel | Aguilera | Adriana | Zepeda | Hospital Country 2000 | Guadalajara | Mexico |
| Sophia | Torres | alejandro | lopez | Hospital de la Familia | Juárez | Mexico |
| Yassine | Hafiani | MOUHAJIR | MOHAMED | CHU IBN SINA | Rabat | Morocco |
| Youns | Chajai | Btissam | El Ghoubar | Cheikh ZAyed Hospital | Rabat | Morocco |
| Aiman | EL FASSI | Amina | Hammour | Institut national d'oncologie | Rabat | Morocco |
| Maria Johana | Barberena Prado | Juan Carlos | Aleman | Hospital Militar | Managua | Nicaragua |
| Mustapha M. | Miko | Abdullahi | Ibrahim | Aminu Kano Teaching Hospital | Kano | Nigeria |
| Saidu Yusuf | YAKUBU | Hauwa | Marut | Ahmadu Bello University Teaching Hospital | Shika-Zaria | Nigeria |
| Vanja | Trajkovska | Biljana | Andonovska | University Clinic of Traumatology, Orthopedic surgery, Anesthesia,Reanimation, Intensive Care and Emergancy department, University Ss Cyril and Methidius | Skopje | North Macedonia |
| Luca | Carenzo | Manuela | Mainetti | IRCCS Humanitas Research Hospital | Rozzano | Italy |
| Ashok | Kumar | Zubaida | Lakhani | Ziauddin university hospital | Karachi | Pakistan |
| Saima | Rashid | Hammad | Yousaf | Aga Khan University Hospital | Karachi | Pakistan |
| Zunairah | Rais | Zunairah | Rais | Liaquat National Hospital | karachi | Pakistan |
| Oscar | Gomez | Kenneth | Ramirez | Hospital Nacional “Dos de Mayo” | Lima | Peru |
| Maria Erika | Ramirez | Flordeliza | Baluyut | St Luke's Medical Center Global City | Taguig City | Philippines |
| Konstanty | Szuldrzynski | Sylwia | Kujawska | National Institute of Medicine of the Ministry of Interior and Administration | Warsaw | Poland |
| Krzysztof | Pietrzkiewicz | Katarzyna | Górecka | Szpital Kliniczny im. K. Jonschera Uniwersytetu Medycznego im. K. Marcinkowskiego | Poznan | Poland |
| Dariusz | Onichimowski | Anna | Antoszewska | Regional Specialist Hospital in Olsztyn | Olsztyn | Poland |
| Cristina | Camilo | Rafael | Marques | Hospital de Santa Maria - ULS Santa Maria | Lisbon | Portugal |
| Gabriela | Almeida | Catarina | Aveiro | Unidade Local de Saúde de Coimbra | Coimbra | Portugal |
| Francisco | das Neves Coelho | Filipa | Cristovao | Unidade Local de Saude de Lisboa Ocidental, Hospital Egas Moniz | Lisboa | Portugal |
| ALEXANDRA | DINIS | SONIA | AZEVEDO | Hospital Pediátrico | Coimbra | Portugal |
| Miguel | Castelo-Branco | Arminda | Pinto | Hospital Pero da Covilha - Unidade Local de Saude da Cova da Beira | Covilha | Portugal |
| Gonçalo | Magalhães | Marta | Soares | Hospital da Luz Lisboa | Lisboa | Portugal |
| José Manuel | Pereira | Marlene | Coelho | Unidade Local Saude S. João | Porto | Portugal |
| EDUARDA | PEREIRA | NOELMA | PEDROSA | CENTRO HOSPITALAR UNIVERSITÁRIO SÃO JOÃO | Porto | Portugal |
| Sara Cristina da Costa | Pereira | Rui | Carneiro | Unidade Local de Saúde do Medio Ave | Vila Nova de Famalicão | Portugal |
| Ion | Chesov | Severin | Ghenadi | Gheorghe Paladi Chisinau City Clinical Hospital | Chisinau | Republic of Moldova |
| GLORIA | RODRIGUEZ VEGA | Arlene | Rivera Maldonado | Sistema de Salud Menonita | Caguas | Puerto Rico |
| Ovidiu | Bedreag | Dan | Ilincariu | ”Pius Brinzeu” Clinical County Hospital Timisoara | Timisoara | Romania |
| Dana | Tomescu | Maria | Manea | Fundeni Clinical Institute | Bucharest | Romania |
| Mircea | Stoian | Colcer | Anca | COUNTY HOSPITAL TIRGU MURES | TIRGU MURES | Romania |
| Janos | Szederjesi | Lazar | Alexandra | Emergency Clinical County Hospital | TÎRGU MUREȘ | Romania |
| Ayyaz | Hussain | Anna | Bogdankova | First city hospital named after E. E. Volosevich | Arkhangelsk | Russian Federation |
| Samiyah | Alanazi | Samiyah | Alanazi | MNGHA | RIYADH | Saudi Arabia |
| Fadi | Aljamaan | Fadi | Aljamaan | King Saud university | Riyadh | Saudi Arabia |
| Mohamed | Kabbani | Kabbani | Mohamed | King Abdulaziz medical city | Riyadh | Saudi Arabia |
| Ahmed | Abdelmaguid | Karim | Jalal | Dr Hamid Suleiman alahmady hospital | Madina | Saudi Arabia |
| Fawziah | Alkhaldi |  | Dr Jamil | KFSHRC | Riyadh | Saudi Arabia |
| Assem | Rezk | Mohammed | Alsayad | Qassim national hospital | Burridah | Saudi Arabia |
| Rawah | Aljishi | Hanan | Ameril | King Salman Hospital | Riyadh | Saudi arabia |
| Hany | Elshouny | jijo | Neyyan | Makkah Medical Centre | Makkah | Saudi Arabia |
| Mirjana | Kačar | Marina | Radovanovic | University clinical center of Serbia | Belgrade | Serbia |
| Mayank | Dalakoti | Shan Nee | Khoo | National University Heart Centre | Singapore | Singapore |
| Natasa | Milivojevic | Diana | Ermenyi | University Medical Centre Ljubljana | Ljubljana | Slovenia |
| Andreja | Möller Petrun | Saüa | Verdnik | University Medical Centre Maribor | Maribor | Slovenia |
| Rendani | Tshitangano | Rendani | Tshitangano | ChrisnHani Bara Academic Hospital | Johannesburg | South Africa |
| Luisa | Charco | Maria Jose | Villora | Hospital General Universitario de Albacete | Albacete | Spain |
| ALVARO | CASTELLANOS-ORTEGA | VERONICA | VIERA | HOSPITAL UNIVERSITARIO Y POLITÉCNICO LA FE | VALENCIA | Spain |
| Pedro | Castro | Inmaculada | Carmona | Clínic Barcelona | Barcelona | Spain |
| Maria Azucena | Pajares | Clara | Marchesi | La Fe | Valencia | Spain |
| Vega | Murga | Rocio | Alonso | CAUSA | Salamanca | Spain |
| Iolanda | Jordan | Marta | Cubells | Hospital Sant Joan de Déu | Barcelona | Spain |
| Pablo | Vidal-Cortés | Estela | Cid Delgado | HU Ourense | Ourense | Spain |
| Francisco Luis | Pérez Caballero | Pilar | Mancha Parejo | Hospital de Mérida | Mérdia | Spain |
| Jorge | Moisés | Montserrat | Medina | Hospital Clínic de Barcelona | Barcelona | Spain |
| María Dolores | Casado Mansilla | Rosario | Solano Martinez | Hospital Santa Lucía | Cartagena | Spain |
| Lucrecia | Blasco | Alicia | Huerta | Consorcio Hospital General Universitario | València | Spain |
| Alvar | Santa Cruz Hernando | Rocío | Sánchez Gómez | Hospital Clínico San Carlos | Madrid | Spain |
| EVA | MANTEIGA RIESTRA | ROBERTO | GOMEZ SANTOS | HOSPITAL INFANTA CRISTINA | PARLA (MADRID) | Spain |
| Maria Pilar | Gracia Arnillas | Marta | Reyes | Hospital del Mar | BARCELONA | Spain |
| Lucía | López Amor | Marta | Nieto Menéndez | Hospital Universitario San Agustín | Avilés | Spain |
| Javier | Pilar | Aitziber | Macoago | Cruces University Hospital | Barakaldi | Spain |
| Eva | Regidor | Ainara | Martorell | Hospital Universitario de Navarra | Pamplona | Spain |
| PATRICIA | JIMENO CUBERO | LUCIA | DIEZ YAGUE | COMPLEJO ASISTENCIAL DE SEGOVIA | SEGOVIA | Spain |
| Sascha | David | Eva-Maria | Kleinert | University Hospital Zurich | Zurich | Switzerland |
| Matthias | Haenggi | Matthias | Haenggi | University Hospital Zurich | Zurich | Switzerland |
| HSIAO-CHENG | CHANG | Hui-Hsin | Huang | Cathay General Hospital | Taipei | Taiwan |
| Nai-Kuan | CHOU | Yung | Yun-Wen | national Taiwan University Hospital | Taipei | Taiwan |
| Hamis | Shafii | Dorcas | Magawa | Muhimbili Orthopedic Institute | Dar es salaam | Tanzania |
| Mohamed | Boussarsar | Salma | Gallas | Farhat Hached University Hospital | Sousse | Tunisia |
| Nacef | BEN MRAD | Soumaya | BEN DHAOU | Abderrahmane Mami | Ariana | Tunisia |
| Didem | Sozutek Akkoyun | Nuray | Kan | Adana city hospital | Adana | Turkey |
| murat | sungur | tugba | demir | erciyes university hospital | kayseri | Turkey |
| Hasan Kutluk | Pampal | Esra | Karataş | Gazi University | Ankara | Turkey |
| Selin | Eyüpoğlu | Arzu | Yücetepe | Ordu State Hospital | Ordu | Turkey |
| Özlem | Özkan Kuşcu | Melisa | Yildiz | Başkent University Adana Dr Turgut Noyan Application and Research Center | Adana | Turkey |
| ipek | erus | nihan | tiryakioglu | Koç University School of Medicine | istanbul | Turkey |
| Oktay | Demirkiran | Bilgi | Cakmak | Istanbul University-Cerrahpasa, Cerrahpasa Murat Dilmener Hospital | Istanbul | Turkey |
| Betul | Yıldız Üstün | Samet | Yazir | Lütfi Kırdar city hospital | İstanbul | Turkey |
| KÜRŞAT | DİKMEN | ASLI EMİNE | BÜYÜKKASAP | Gazi University Hospital | Ankara | Turkey |
| Sinem | Keskin Kayalar | Yeşim | Selçuk | Health Science University | İstanbul | Turkey |
| erdem | yalçınkaya | cagla | sahin | marmara university | istanbul | Turkey |
| ahmet | cosar | kübra | çalışkan | Gülhane Hastanesi | Ankara | Turkey |
| Mustafa | Ay | Hasan | Dogan | Antalya research and training hospital | Antalya | Turkey |
| dilara | tüfek öztan | esvet | inanoğlu | Antalya research and traıning hospital | Antalya | Turkey |
| Handan | Birbicer | Aynur | Ekici | Mersin University Faculty of Medicine | MERSİN | Turkey |
| YELDA | BALIK | TUNA | KARAHAN | ISTANBUL HAYDARPASA NUMUNE TRAINING HOSPITAL | ISTANBUL | Turkey |
| Hossam | ElshekhAli | Ragab | Abdelhady | NMC Royal | Abu Dhabi | United Arab Emirates |
| alaaeldien | ghanem | Anu | Peter | Khorfakan | Sharjah | United Arab Emirates |
| Mohamed Anwar Abdelsalam Mohamed | Mohamed | Enriqueta | Bernas | NMC Specialty Hospital | Al Ain, Abu Dhabi | United Arab Emirates |
| Janine | Lonsdale | Eleanor | Higgs | City Hospital | Birmingham | UK |
| Yayati | Joshi | Linda | Pipira | Bristol royal infirmary | Bristol | UK |
| Kali | Cann | Tina | Wright | Kings Mill Hospital | Mansfield | UK |
| Nikhail | Balani | Rebecca | Seaman | Maidstone and Tunbridge Wells Hospitals | Maidstone | UK |
| Priya | George | Mary | Thompson | Cheltnenham hospital | Cheltenham | UK |
| Campbell | Tarni | Gardner | Hollie | Crosshouse hospital | Kilmarnock | UK |
| Ravishankar | Nagaraj | Claire | Jennings | Royal Manchester Children's Hospital | Manchester | UK |
| Simon | Finney | Rosalie | Magboo | St Bartholomew's Hospital | London | UK |
| LIU | XIAOYI | Gellmaucho | Minnie | University Hospitals of North Midlands NHS Trust | Royal Stoke University Hospital | UK |
| MOSES | CHIKUNGWA | Minnie | Gellamucho | UNIVERSITY HOSPITALS OF NORTH MIDLANDS NHS TRUST | STOKE ON TRENT | UK |
| Kateryna | Bielka | Semenko | Natalia | University clinic | Kyiv | Ukraine |
| Lucciano | Grasiuso | Mónica | Coirolo | CAMEDUR | Durazno | Uruguay |
| Mariana | Noya | Ignacio | Trujillo | Asociación Médica de San Jose | San Jose | Uruguay |
| Gaston | Burghi | Luciana | Vasallo | Hospital Evangélico | Montevideo | Uruguay |
| Mariana | Noya | Ignacio | Trujillo | Asociación Médica de San Jose | San Jose | Uruguay |
| Ignacio | Aramendi | lourdes | Ferro | Hospital de clínicas | Montevideo | Uruguay |
| Gaston | Burghi | Analia | Martins | Hospital Maciel | Montevideo | Uruguay |
| ibrahim | al-sanouri | Nicole | Morgan | Ibrahim AL Sanouri (McLaren Flint Hospital) | Troy Michigan | USA |
| Ranier | Reyes | Meera | Viswanathan | University of Texas Southwestern Medical Center | Dallas | USA |
| Ronald | Cardenal | Leidy | Bolivar | Hospital Privado Centro médico de Caracas | Caracas | Venezuela |
| Andrew | Turner | Ellen | Burke | Royal Hobart Hospital | Hobart | Australia |
| QUENTIN | LEVRAT | QUENTIN | LEVRAT | Groupe Hospitalier de La Rochelle | La Rochelle | France |
| Rene | Schmutz | Andrea | Schmidt | Vienna General Hospital | Vienna | Austria |
| Erwin | Grasmuk-Siegl | Elke | Vas | Klinik Floridsdorf | Vienna | Austria |
| Pierre | Raeven | Andrea | Kunert | Vienna General Hospital | Vienna | Austria |
| Fritz | Firlinger | Angelika | Kuhn | Konventhospital Barmherzige Brüder | Linz | Austria |
| Thomas | Eisner | Claudia | Willheim | University Hospital Tulln | Tulln | Austria |
| Abu Hena Mostafa | Kamal | Tanjina | Islam | Rajshahi Medical College Hospital | Rajshahi | Bangladesh |
| Joop | Jonckheer | Evelien | Spruyt | Universitair Ziekenhuis Brussel | Jette | Belgium |
| Edin | Kabil | Kadrija | Kadrovic | Cardiovascular surgery, Clinical center University of Sarajevo | Sarajevo | Bosnia and Herzegovina |
| Aida | Coric | Oksana | Memic | JU KB Dr.Safet Mujic | Mostar | Bosnia and Herzegovina |
| Eduardo | Minuzzo | Aline | Barbosa | Cooperar Hospital | Vilhena | Brazil |
| MARIANNA | PONTES | MARIANNA | PONTES | Real Hospital Português de Beneficencia | Recife | Brazil |
| Ahmad | Alli | Kinza | Malik | St Michael's hospital | Toronto | Canada |
| Fernando | Tirapegui | Gladys | Jara | Complejo Asistencial Dr. Víctor Ríos Ruiz | Los Angeles | Chile |
| MONICA | SALINAS | ALEJANDRA | PIZARRO | HOSPITAL DE NIÑOS DOCTOR EXEQUIEL GONZALEZ CORTES | SANTIAGO | Chile |
| PAUL | SANTILLAN | DIEGO | PALLERO | HOSPITAL CLINICO VIÑA DEL MAR | VIÑA DEL MAR | Chile |
| Gavin | Joynt | Carmen | Ho | Prince of Wales | Hong Kong | China |
| Yifei | Chen | Bingchao | Xu | Affiliated Hospital of Yangzhou University | Yangzhou | China |
| JUAN | GUTIERREZ | Luz Stella | Montoya | CLÍNICA LOS NEVADOS | PEREIRA | Colombia |
| Renato | Castaño | D. F. | Cardenas | Clínica Medilaser | Neiva | Colombia |
| Dora | Karmelić | Ante | Santini | UHC Zagreb | Zagreb | Croatia |
| Jasminka | Persec | Tomislava | Gospocic | UH Dubrava | Zagreb | Croatia |
| Sonja | Krofak | Petra | Vrbnjak | University hospital Sveti Duh | Zagreb | Croatia |
| Pablo | Morocho | Lesly | Rodriguez | Hospital de Especialidades Portoviejo | Portoviejo | Ecuador |
| Aiman | Al-Touny | Aiman | Al-Touny | Suez Canal University | Ismailia | Egypt |
| Mary Christeen | Sharobeem | Hala | Soliman | Sharq El Madina Hospital | Alexandria | Egypt |
| Valdo | Toome | Andra-Maris | Post | NorthEstonia Medical Centre | Tallinn | Estonia |
| Diriba | Fayisa | Elias | Oljira | Wallaga University Comprehensive Specialized Hospital | Nekemte | Ethiopia |
| Tekiy Markos | Bedore | Truelove | Ahmed temesgen | Wachemo university Nigist Ellini Mohammad Memorial Hospital | Hosana | Ethiopia |
| Yemane | Tesfay | Ephrem | Ahmed | St. Paul's Hospital Millennium Medical College | Addis Ababa | Ethiopia |
| Laetitia | Bodet-Contentin | Agnès | Clement | CHU de Tours - Hopital Bretonneau | Tours | France |
| Guillaume | Dumas | Priscillia | Gamon | CHU MICHALLON | Grenoble | France |
| Alexandra | Beurton | Alexandra | BEURTON | Tenon APHP | Paris | France |
| LOIC | DOPEUX | MARINE | TAVARES | CH Lacarin | Vichy | France |
| Eddy | Lebas | Sébastien | Bigot | Centre Hospitalier Bretagne Atlantique | VANNES | France |
| Pierre-Yves | Delannoy | Vanessa | Gueville | Guy Chatillez | Tourcoing | France |
| Vivien | HONG TUAN HA | Catherine | LELONG | Grand Hopital de l'Est Francilien | Meaux | France |
| Olfa | Hamzaoui | Maxime | Rocquet | Robert Debré | Reims | France |
| JULIO | BADIE | Nahila | HIMER | HOPITAL NORD FRANCHE COMTE | TREVENANS | France |
| Aurélie | LEJEUNE | Aurelie | Lejeune | Centre Hospitalier de Périgueux | Périgueux | France |
| Rachele | Conti | Brice | Pellegrini | Hôpital du pays Salonais | Salon de Provence | France |
| Philippe | GAUDARD | Myriam | NOMDEDEU | Arnaud de Villeneuve Hospital, CHU Montpellier | Montpellier | France |
| Dorothée | Campos | Mélanie | Naud | Centre Hospitalier Loire Vendée Océan | Challans | France |
| EMMANUEL | FUTIER | PERINE | VIDAL | Estaing Hospital | Clermont-Ferrand | France |
| Frank | Vogel | Zlatan | Pejic | Artemed Klinikum München Süd | Munich | Germany |
| Sebastian | Brenner | Frida | Regner | Paediatric Intensiv Care Unit, Department of Pediatrics, University Hospital Carl Gustav Carus, TU Dresden | Dresden | Germany |
| Johannes | Gramatté | Marco | Reinhardt | University Hospital Carl Gustav Carus at the TU Dresden | Dresden | Germany |
| Akwasi | Antwi-Kusi | Akwasi | Antwi-Kusi | Komfo Anokye Teaching Hospital | Kumasi | Ghana |
| Hamish | Thomson | Raquel | Piner | St Bernard's | Gibraltar | Gibraltar |
| ALEXANDRA | PENTHEROUDAKI | Olga | Maderaki | ST Georges General Hospital of Chania | Chania | Greece |
| Maria | Theodorakopoulou | Sophia | Dimitriou | General Hospital of Attiki KAT | Athens | Greece |
| Frantzeska | Frantzeskaki | OLGA | GATZIA | ATTIKON UNIVERSITY HOSPITAL | ATHENS | Greece |
| Eva-Otilia | Nagy | Koutsouki | Sotiria | General Hospital of Kavala | Kavala | Greece |
| Aikaterini | Sakagianni | Georgia | Makariti | Sismanogleio General Hospital | Maroussi | Greece |
| Athina | Lavrentieva | Anastasia | Bikouli | Papanikolaou Hospital | Thessaloniki | Greece |
| Aikaterini | Flevari | George | Kourtis | Thoracic Diseases General Hospital "Sotiria" | Athens | Greece |
| Georgios | Koukoulitsios | Nadezda | Panagiotopoulou | General Hospital of Athens ''G Gennimatas'' | Athens | Greece |
| MARIA | KAZANTZI | MARIA | TSOUTSI | "AGHIA SOFIA" CHILDREN'S HOSPITAL | ATHENS | Greece |
| Fotios | Tsimpoukas | Aglaia | Vlachaki | Lamia General Hospital | Lamia | Greece |
| GEORGE | BINIARIS | ARISTEA | PETSA | METAXA ANTICANCER HOSPITAL | PIREAUS | Greece |
| Laszlo | Zubek | Réka | Jung | Semmelweis Univesity | Budapest | Hungary |
| Adam | Peter | Timea | Talas | Észak-pesti Centrumkórház - Honvédkórház / Central Hospital of Northern Pest - Military Hospital | Budapest | Hungary |
| Parveen | Kaur | Anchal | Batra | Rajiv Gandhi cancer hospital and research centre. | New Delhi | India |
| venkateswaran | sundararajan | tanya | ann mcgregor | manipal hospital | bangalore | india |
| GURURAJ | GUNDA | LALITHA | KIRAN | KAUVERY HOSPITAL | BENGALURU | India |
| Berdai | adnane |  |  |  |  | Morocco |
| Christopher | Mathew | Manish | Murali | Dr.Moopens Medical College Hospital | Wayanad | India |
| Amrit | Kaur | Abhaya | Mahadik | Dr.vasantrao pawar hospital and research centre | Nashik | India |
| sandeep | mangla | Jiteender | Singh Shekhawat | Amrita institute of medical sciences | FARIDABAD | India |
| KESHAV | POCHAM | Yellam | Lacchaiah | Kims hospital | Hyderabad | India |
| Yash | Javeri | yash | javeri | Regency Hospital | lucknow | India |
| Vijay | Sundarsingh | Sheral | Crasta | Father muller Medical college hospital | Mangalore | India |
| Akshay | HM | Mala | N | JSS Hospital | Mysore | India |
| Nita | George | Treesa | Brice | VPS Lakeshore Hospital | Kochi | India |
| Neeraj | Kumar | Sunita | Kachhap | All India Institute of Medical Sciences Patna | Patna | India |
| Aswani Kumar | Balakrishna Pillai | Vismaya | Puthen purayil | Nahas hospital | Parappanangadi | India |
| reddy | Madhava | muthangachalil | jisha | yashoda hospital - hitech city | hyderabad | india |
| Vineeta | Goyal | Karthika | K | Kailash hospital and Neuro institute | Noida | India |
| Nagarajan | Ramakrishnan | C V | Sheela | Apollo Main Hospital | Chennai | India |
| Kandaswamy | N | Amirthavalli | Athimoolam | Jawaharlal Institute of Postgraduate Medical Education and Research(JIPMER) | Puducherry | India |
| PAYEL | BOSE | MOHAMMED | RAFI | MEDICA SUPERSPECIALTY HOSPITAL | Kolkata | India |
| Sara | Vergis | Jinny | Joseph | MOSC MEDICAL COLLEGE | Ernakulam | India |
| Dr ziyokov | Joshi | Joshi | Atul | Tagore hospital and heart care | Jalandhar | India |
| Abhinav | Lambe | Abhinav | Lambe | Pravara Hospital | loni | India |
| Gopala Krishnan | Ravi | Vanitha | Sahadevan | Manipal Northside hospital | Bangalore | India |
| DEEPAK | GOVIL | SHIJI | ANTONY | MEDANTA, THE MEDICITY | GURGAON | India |
| Harcharan | Kaur | Manpreet | Kooner | Deep Hospital | Ludhiana | India |
| Seyed MohammadReza | HASHEMIAN | Iraj | Fotohi | Masih Daneshvari Hospital | Tehran | Iran |
| hayder | al-kuraishy | rania | majed | al-yarmmok teaching hospital | Baghdad | Iraq |
| Aras | Sediq | Widyan | Mirza | Azadi teaching hospital | Dahuk | Iraq |
| Sinead | Farrell | Lorraine | Convey | Sligo University Hospital | Sligo | Ireland |
| Josephine | Kelliher | Serena | O'Brien | Mater Misericordiae University Hospital | Dublin | Ireland |
| Sinead | Egan | Fiona | O'Shea | Mayo University Hospital | Castlebar | Ireland |
| Roy | Ilan | Osnat | Aspis | Rambam health care center | Haifa | Israel |
| Moran | Hellerman Itzhaki | Anna | Kofman | Rabin medical center | Petah Tikva | Israel |
| Vernon | van Heerden | Shelly | Ashkenazi | Hadassah University Hospital | Jerusalem | Israel |
| Neta | Golomb | Ahmed | Blawona | Tzafon Medical Center | Poriya | Israel |
| Giovanni | Mistraletti | Laura | Merlini | Ospedale Civile di Legnano | Legnano, MI | Italy |
| Vincenzo Francesco | Tripodi | Ivan | Alonge | "Gaetano Martino" University Hospital | Messina | Italy |
| Vittorio | Ferrari | Giulia | Bombini | Istituto ortopedico rizzoli | Bologna | Italy |
| Mariachiara | Ippolito | Pasquale | Iozzo | Azienda ospedaliera policlinico Paolo giaccone | Palermo | Italy |
| KATIA | DONADELLO | ELENA | SASSI | UNIVERSITY HOSPITAL INTEGRATED TRUST OF VERONA | VERONA | Italy |
| Fullin | Giorgio | Mauriello | Chiara | Ca Foncello | Treviso | Italy |
| Plinio | Calligaro | Daniela | Braghiroli | Ospedale Magalini | Villafranca di Verona | Italy |
| Carlo | Olivieri | Biagio | Santoro | Sant'Andrea Hospital | Vercelli | Italy |
| Conio | Alessandra | Bergese | Ilaria | Ospedale infantile Regina Margherita | Torino | Italy |
| Caterina Federica | Vassallo | Roberta | Garavello | San Giovanni Bosco | Torino | Italy |
| Cecilia | Pagano | Cecilia | Pagano | Mauriziano Hospital | Torino | Italy |
| Abdulrahman | Al-Fares | Manahel | Sallom | Amiri Hospital | Kuwait City | Kuwait |
| Sarah | Buabbas | Anu | James | Jaber Al-Ahmad alsabah hosptial | Kuwait | Kuwait |
| Abdurraouf | Abusalama | mohammed omar | Abdulhade | Preventive Medicine Hospital | Tripoli | Libya |
| Ausra | Puziene | Laura | Dygle | The Hospital of Lithuanian University of Health Sciences | Kaunas | Lithuania |
| LUIS ALEJANDRO | SANCHEZ-HURTADO | VIRGINIA | MARTINEZ-ENRIQUEZ | UMAE Hospital de Especialidades "Bernardo Sepulveda Gutierrez" Centro Medico Nacional Siglo XXI IMSS | Mexico city | Mexico |
| ALFREDO | ARELLANO | ricardo | Osnaya | Hospital regional de alta especialidad ixtapaluca | Mexico | Mexico |
| SOUMAYA | TOUZANI | HAMID | GUEDDAH | HASSAN II UNIVERSITY HOSPITAL | FEZ | Morocco |
| FAISAL | AMEZIANE EL HASSANI | FAISAL | AMEZIANE EL HASSANI | Hôpital Hassan II Service de Réanimation | SETTAT | Morocco |
| OUISSAL | AISSAOUI | NAJLAA | ELBAHI | Abderrahim Harouchi Mother-Child hospital | Casablanca | Morocco |
| Tarek | DENDANE | Tarek | DENDANE | Ibn Sina Hospital | Rabat | Morocco |
| My Ahmed | Bouderka | Cherragi | Karima | Dar Salam clinic | Casablanca | Morocco |
| Job | Dr Otokwala | Otonye | Derefaka | University of Portharcourt teaching hospital | Portharcourt | Nigeria |
| Bilquees | Saba | bilquees | Saba | Ziauddin university hospital | Karachi | Pakistan |
| Abdul | Majid | Ali | Jan | Jinnah postgraduate medical center | Karachi | Pakistan |
| Juan Luis | Pinedo Portilla | Diana | Coronel Guevara | Clínica AUNA | Chiclayo | Peru |
| TOMMY | PRADO | GLORIA | GOMEZ | INSTITUTO NACIONAL CARDIOVASCULAR CARLOS ALBERTO PESCHIERA CARRILLO | LIMA | Peru |
| Agnieszka | Wieczorek | Paulina | Koziel | 4th Military Clinical Hospital with Polyclinic SPZOZ in Wrocław | Wrocław | Poland |
| Patricia | Patricio | Joana | Valente | Hospital da Luz Setúbal | Setúbal | Portugal |
| Ramiro | Carvalho | Fernanda | Conceição | Champalimaud Foundation | Lisbon | Portugal |
| Ricardo | Matos | Filomena | Sanches | Nossa Senhora do Rosário | Barreiro | Portugal |
| Carla | Teixeira | Helena | Bessa | Centro Hospitalar Universitário de Santo António | Porto | Portugal |
| Bessa de Melo | Mafalda | Pinto | Cristina | Hospital Padre Américo | Penafiel | Portugal |
| Juliana | Mortagua | Daniela | Plácido | Hospital São José | Lisbon | Portugal |
| Filipa | Almeida | Elisabete | Figueiredo | Hospital de São Teotónio-Viseu | Viseu | Portugal |
| Diogo | Oliveira | Sofia | Rodrigues | ULSTMAD | Vila Real | Portugal |
| Anca Irina | Ristescu | Patrascanu | Emilia | Regional Institute of Oncology | Iasi | Romania |
| Cosmin | Balan | Irina | Stoian | CC Iliescu Emergency Institute for Cardiovascular Diseases | Bucharest | Romania |
| Khairallah | Belkhouja | Sarah | Almazwaghi | King Fahd Armed Forced Hospital (KFAFH) | Jeddah | Saudi Arabia |
| Ahmed | Mady | Hanan | Alonazi | King Saud Medical City | Riyadh City | Saudi Arabia |
| Ayman | Kharaba | Ahmad | Alsaedi | King fahad hospital | Madinah | Saudi Arabia |
| Yaseen | Arabi | Samiyah | Alanazi | MNGHA | RIYADH | Saudi Arabia |
| GHALEB | ALMEKHLAFI | HAIFA | HARUN | KING SALMAN BIN ABDULAZIZ MEDICAL CITY-MAIN HOSPITAL | MADINAH | Saudi Arabia |
| Srđan | Gavrilović | Maja | Stanojevic | Institute for pulmonary diseases of Vojvodina | Sremska Kamenica | Serbia |
| Matthew | Cove | Zauyeh | Sanif | National University Hospital | Signapore | Singapore |
| Andrej | Markota | Jasmina | Golenko | University Medical Centre Maribor | Maribor | Slovenia |
| Maria Dolores | Bosque | Silvia | Pilar | Hospital Universitari General de Catalunya | Barcelona | Spain |
| Elena | Martínez | Ana Rosa | Alonso | Morales Meseguer | Murcia | Spain |
| Irene | Pérez Blanco | Ana | Oblanca | CAULE | Leon | Spain |
| Angel | Pobo | Diana | Gil | Joan XXIII University Hospital | Tarragona | Spain |
| Maricela | Jiménez-López | Marina | Parrado Aguilar | Hospital de Tortosa Virgen de la Cinta | Tortosa, Tarragona | Spain |
| Álvaro | Ortega Guerrero | Marta | Fernandez Laguna | Hospital Quironsalud Málaga | Malaga | Spain |
| Gabriel | Heras La Calle | Josefina | Morales Rodríguez | Hospital Universitario de Jaén | Jaén | Spain |
| Pablo | Rama-Maceiras | Isabel | Vazquez-Castelos | Hospital Universitario A Coruña | A Coruña | Spain |
| Carlos | Seron Arbeloa | Sandra | Bescos Vaquero | Hospital Universitario San Jorge | Huesca | Spain |
| Maria Carmen | Sala-Trull | Lidia | Carrera | Clinica Universidad de Navarra | Pamplona | Spain |
| Maria Cruz | Martin Delgado | Frade Mera | Maria Jesús | Hospital Universitario 12 Octubre (Polivalente) | Madrid | Spain |
| Maria Cruz | Martin | Maria del Ara | Murillo | Hospital Universitario 12 Octubre (UCITE) | Madrid | Spain |
| Alejandra | Fernández Trujillo | Mireia | Sánchez Anguita | Hospital Universitari Parc Taulí | Sabadell, Barcelona | Spain |
| Antonio | Ferraroni | Elisabet | Ferrando Sanchez | Hospital del Mar | Barcelona | Spain |
| Paola Beatriz | Vergara Diaz | Laura | Pérez-Abadín Barro | Hospital Universitario Central de Asturias | Oviedo | Spain |
| Adriana | Jacas Coma | Adriana | Ruiz | Hospital Clinic de Barcelona | Barcelona | Spain |
| Luis | Morales-Quinteros | Laura | Vidal | Vall d'Hebron University Hospital | Barcelona | Spain |
| Ivette | Chocron | Concepción | Vazquez | Hospital Vall d’Hebron | Barcelona | Spain |
| Govind | Sridharan | Joel | Scheder | Hôpital fribourgeois | Fribourg | Switzerland |
| Hannah | Wozniak | Laetitia | Langlois | Geneva University Hospitals | Geneva | Switzerland |
| Maria-helena | Perez | Chantal | Grandjean | University Hospital of Lausanne | Lausanne | Switzerland |
| Shelley | Tsai | Shelley | Tsai | national taiwan university hospital | taipei | Taiwan |
| Amel | MOKLINE | Dekhra | Sliti | Trauma and burn center | Tunis | Tunisia |
| Fatma | Essafi | Fatma | Essafi | Zaghouan | Zaghouan | Tunisia |
| Seda | Akinci | duygu | dölen | hacettepe üniversitesi erişkin hastanesi | Ankara | Turkey |
| Ozkul Yilmaz | Colak | Aysun | Gokce | Ondokuz Mayis University, Faculty of Medicine Hospital | Samsun | Turkey |
| Aycan | Göktürk | Belgin | Tavşan | Istanbyl University- Cerrahpaşa Prof Dr Murat Dilmener hospital | Istanbul | Turkey |
| Nazim | Dogan | Tuğçe | Akın | Atatürk University Medical School, Department of Anestehesiology, ICU | Erzurum | Turkey |
| Özgür | Kılıç | Melda | İsevi | 19 Mayıs üniversitesi | Samsun | Turkey |
| AYCA | GUMUS | CETIN | EMINE | KEPEZ STATE HOSPITAL | ANTALYA | Turkey |
| Ayse Belin | OZER | İlayda | DAGDELEN | Inonu University Turgut Ozal Medical Center | Malatya | Turkey |
| Kutlay | AYDIN | Bircan | AKI | TURKISH MINISTRY OF HEALT AYDIN STATE HOSPITAL | Efeler/AYDIN | Turkey |
| Duygu | Kayar Calili | Ata Evren | Tike | Ankara Bilkent City Hospital | Ankara | Turkey |
| Gulsum | Altuntas | Gulsum | Altuntas | Firat University Medicine Faculty Hospital | Elazig | Turkey |
| Ayşe | Çapar | Özlem | Aygün | İstanbul Sultan Abdulhamid Han Training and Research Hospital | İstanbul | Turkey |
| KAMURAN | ULUÇ | HAYAT | INCE | MUŞ STATE HOSPİTAL | MUŞ | Turkey |
| Mehmet | Kilic | Mehmet | Kilic | Van Yüzüncü Yıl University Dursun Odabaş Medical Center | Van | Turkey |
| Jane | Nakibuuka | John | Osire | Mulago National Referral Hospital | Kampala | Uganda |
| Monica | jadhav | Monica | jadhav | NMC Specialty Hospital, Al Ain | Al Ain, Abu Dhabi | United Arab Emirates |
| Dr Nitin | Tarale | Sumble | Raza | NMC ROYAL HOSPITAL | Sharjah | United Arab Emirates |
| Dr. Sunil | Garg | Amit kumar | Kumawat | NMC Royal Hospital | Dubai | United Arab Emirates |
| Hossam | ElshekhAli | Ragab | Abdelhady | NMC Royal | Abu Dhabi | United Arab Emirates |
| Rachael | Fieldhouse | Nicholas | Taylor | Queen's Medical Centre | Nottingham | UK |
| Lisa | Halsall | Lisa | Halsall | Royal Preston Hospital | Preston | UK |
| Daniel | Harvey | Rachael | Fieldhouse | Queens Medical Centre | Nottingham | UK |
| Carole | Boulanger | Helen | Baker | Royal Devon NHS University Foundation Trust | Exeter | UK |
| Turton | Peter | Ingram | Alexandra | Warrington and Halton Hospitals NHS Foundation Trust | Warrington | UK |
| Caroline | Lacey | Teresa | Scott | Aberdeen Royal Infirmary | Aberdeen | UK |
| Mason | Sabina | NADER | AL-MANE |  | Co. Kildare | Ireland |
| Francesco | Vasques | Linda | Tovey | Guy’s and St Thomas’s NHS Foundation Trust | London | UK |
| James | Park | Ma Zeycel Roxanne | Dasig | Freeman Hospital | Newcastle upon Tyne | UK |
| Fiona | Lamb | Jackie | Mitchell | East Surrey Hospital | Redhill | UK |
| Priya | George | Debbie | Seal | Gloucester royal hospital | Gloucester | UK |
| anita | jones | sonia | obiano | Russells Hall Hospital | Dudley | UK |
| Helen | McMillan | Liana | Stapleton | University Hospitals Plymouth | Plymouth | UK |
| Brian | Hogan | Amitaa | Maharajh | Royal Free Hospital | London | UK |
| Dominique | Byrne | Kirsty | Edwards | Queen Alexandra hospital | Portsmouth | Uk |
| Lia | Paton | Debbie | McDonald | Glasgow Royal Infirmary | Glasgow | UK |
| Tomasz | Torlinksi | Tomasz | Torlinksi | Queen Elizabeth Hospital, University Hospitals Birmingham | Birmingham | uk |
| Ihor | Yovenko | Kyrylo | Miros | Medical Home Odrex | Odessa | Ukraine |
| Olena | Khomenko | Yelizaveta | Stadnik | National Scientific Center Surgery and Transplantation named by Shalimov | Kyiv | Ukraine |
| Gaston | Burghi | Gabriela | Mendez | Hospital de Clinicas | Montevideo | Uruguay |
| Paula | Genta | Natalia | Saboredo | COMEPA | Paysandú | Uruguay |
| Andreas | Schwingshackl | Shanae | Williams | Mattel Children's Hospital | Los Angeles | USA |
| William | Harding | Kimberly | Barton | Memorial Hermann - Texas Medical Center | Houston, TX | USA |
| Akram | Zaaqoq | Ashley | Wade | University of Virginia | Charlottseville | USA |
| Paul | McCarthy | Alan | Alimario | Ruby Memorial Hospital | Morgantown | USA |
| Peter | Morris | YY | XX | University of Alabama at Birmingham | Birmingham | USA |
| Amy | Lee | Kari | Guffey | Children's Medical Center | Dallas | USA |
| Asma | Zainab | Daniel | Kerr | Houston Methodist Hospital | Houston | USA |
| Rajeev | Patel | Kate | Murphy | Peconic Bay Medical Center | Riverhead | USA |
| Quy | Hoang | Truong | Tran | 108 Military Central Hospital | Ha Noi | Việt Nam |
